# Sensorial Hierarchy in *Octopus vulgaris’s* Food Choice: Chemical vs. Visual

**DOI:** 10.3390/ani10030457

**Published:** 2020-03-10

**Authors:** Valeria Maselli, Al-Sayed Al-Soudy, Maria Buglione, Massimo Aria, Gianluca Polese, Anna Di Cosmo

**Affiliations:** 1 Department of Biology, University of Napoli Federico II, Complesso Universitario Monte Sant’ Angelo, Via Cinthia, 80126 Napoli, Italy; valeria.maselli@unina.it (V.M.); alsayedalsoudymohamed.mostafa@unina.it (A.-S.A.-S.); maria.buglione@unina.it (M.B.); 2 Department of Economics and Statistics, University of Naples Federico II, Complesso Universitario Monte Sant’Angelo, Via Cinthia, 80126 Napoli, Italy; massimo.aria@unina.it

**Keywords:** *Octopus vulgaris*, cephalopod behaviour, problem-solving, cephalopod, chemical cues, visual cues, food choice, octopus sense organs

## Abstract

**Simple Summary:**

Coleoids are cephalopods endowed with a highly sophisticated nervous system with keen sense organs and an exceptionally large brain that includes more than 30 differentiated lobes. Within this group, *Octopus vulgaris,* well known as an intelligent soft-bodied animal, has a significant number of lobes in the nervous system dedicated to decoding and integrating visual, tactile, and chemosensory perceptions. In this study, we aimed to understand the key role of chemical and visual cues during food selection in *O. vulgaris*. We first defined the preferred food, and subsequently, we set up five different problem-solving tasks, in which the animal’s choice is guided by visual and chemosensory signals, either alone or together, to evaluate whether individual *O. vulgaris* uses a sensorial hierarchy. Our behavioural experiments show that this species does integrate different sensory information from chemical and visual cues during food selection; however, our results indicate that chemical perception provides accurate and faster information leading to food choice. This research opens new perspectives on *O. vulgaris*’ predation strategies.

**Abstract:**

*Octopus vulgaris* possesses highly sophisticated sense organs, processed by the nervous system to generate appropriate behaviours such as finding food, avoiding predators, identifying conspecifics, and locating suitable habitat. Octopus uses multiple sensory modalities during the searching and selection of food, in particular, the chemosensory and visual cues. Here, we examined food choice in *O. vulgaris* in two ways: (1) We tested octopus’s food preference among three different kinds of food, and established anchovy as the preferred choice (66.67%, Friedman test *p* < 0.05); (2) We exposed octopus to a set of five behavioural experiments in order to establish the sensorial hierarchy in food choice, and to evaluate the performance based on the visual and chemical cues, alone or together. Our data show that *O. vulgaris* integrates sensory information from chemical and visual cues during food choice. Nevertheless, food choice resulted in being more dependent on chemical cues than visual ones (88.9%, Friedman test *p* < 0.05), with a consistent decrease of the time spent identifying the preferred food. These results define the role played by the senses with a sensorial hierarchy in food choice, opening new perspectives on the *O. vulgaris*’ predation strategies in the wild, which until today were considered to rely mainly on visual cues.

## 1. Introduction

The sensory systems of animals are crucial to detect environmental cues, and they are then processed through the nervous system to generate appropriate behaviours [1], such as finding food, avoiding predators, identifying conspecifics, locating suitable habitat, and attracting mates [2,3,4]. In aquatic systems, as on land, chemical cues affect not only individual behaviour and population dynamics, but also community organisation and ecosystem function.

Use of sensory modalities may be related to the ecology of the species, as prey or predator. Animals use different sensory modalities to search for food such as chemical, vibrational, tactile, sound, heat, and visual senses [5]. Among them, while vision enables marine animals to swim directly to food items when they see it, chemoreception is essential to detect and locate food items, especially for animals active at night or in the deep ocean [6,7,8]. Several studies suggest that aquatic species rely more strongly on chemical perception rather than vision one when discriminating between harmless and dangerous heterospecifics [9,10,11,12]. On the other hand, aquatic environments are particularly prone to the variability of the visual and chemical conditions. For example, turbidity could reduce the efficacy of visual cues even if octopus is capable of polarised vision [13,14,15], whereas currents may disrupt chemical information.

Accordingly, the sensory system’s capabilities in cephalopods have been inextricably associated with their evolutionary success, allowing them to occupy many ecological niches of the sea from shallow waters to the deep sea. Coleoids are endowed with a highly sophisticated nervous system [16,17,18] and an exceptionally large brain that includes more than 30 differentiated lobes [17,19]. Among them, *Octopus vulgaris* (hereafter octopus)*,* well known as an intelligent soft-bodied animal, has a significant number of lobes of the nervous system dedicated to visual, tactile, and chemosensory perception [20,21]. Its nervous system has a high degree of cross-connectivity [17,20,22] able to integrate sensory inputs coming from the environment through its well-developed sensory organs [17,23,24,25].

**Figure 1:**
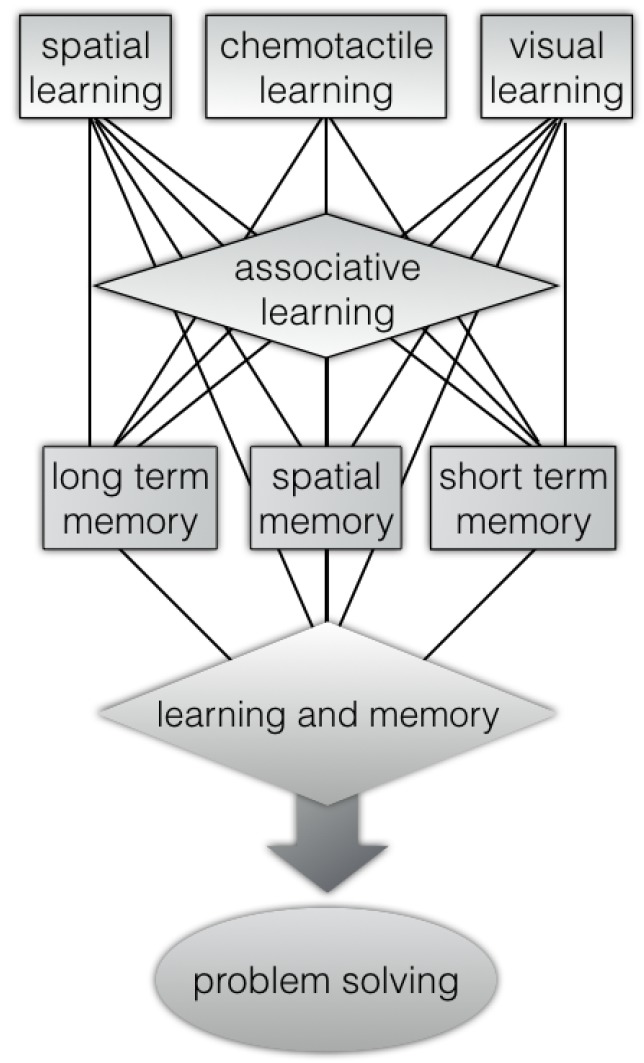
Octopus’s problem-solving abilities [26,27,28,29,30,31,32] through learning and memory abilities [19,33,34,35,36,37,38,39,40,41,42] (associative learning [43,44,45], spatial memory [46,47,48], visual learning [43,49,50,51], chemo-tactile learning [52], long-term and short-term memory [34,38,39,53,54]).

Indeed, octopus has a rich repertoire of complex behaviours (Figure 1) that includes problem-solving, visual, and chemo-tactile.

In particular, the abilities of coleoids to perceive environmental cues have been mainly attributed to its visual systems. Although, under limited light conditions, the chemical signals are the primary important source as sensory inputs [23,55,56,57]. Thus, coleoids have remarkable abilities to recognise chemical cues through the buccal lips and mouth [58], isolated sensory neurons [59,60], and arm suckers [25,61,62,63]. Thus, they may explore their environment by touch and taste, while their olfactory organs are able to perceive at distance [23,55,64,65,66,67,68,69], sensing a broad spectrum of chemical signals [22]. 

It has been reported that chemosensory cues are important in decision-making in octopuses [22,65,66,67,68,69,70,71,72,73]. Training experiments for testing chemical discrimination have been done in octopus to demonstrate its ability to distinguish between objects based on their chemical differences using their arm suckers and described this ability as taste by touch [24,25], while odour discrimination was tested to assess perceptions of water-born chemical stimuli at distance [72]. Furthermore, it has been highlighted that the octopus’s olfactory organ is able to change shape, from relaxed to erect to perceive water-soluble compounds such as salts, sugars, amino acids, amines, peptides, proteins, and functionalised hydrocarbons, which allows the animal to orient itself to detect the spatial gradient of these chemical cues, helping in navigation and triggering spatial memories [23,57,74,75]. Octopuses also possess a self-recognition mechanism, which consists of the attachment reflex inhibition of their own suckers, due to chemical signals in the skin [76]. Recently, it has been hypothesised that olfaction in octopus is not restricted to the olfactory organ, but it is also extended to other structures such as the suckers, that were traditionally not considered olfactive. In particular, octopus exhibits a peculiar performance that can be defined “smell by touch”, useful to detect odorant molecules that in water are insoluble or have a very low solubility [55,74,77,78]. 

However, the octopus has always been described as a predominately “visual” animal with a complex visual system characterised by the presence of highly developed eyes [22,79,80,81,82]. Analogously to vertebrate, octopus eyes are equipped with an un-inverted retina, a cornea, an iris, and a lens. Even if they have just one type of receptor cell and only rhodopsin as pigment, octopuses have the ability to recognise the plane of polarised light based on rhabdomeres dichroism. Moreover, it has been proposed that they are able to discriminate colours [15] within a wide range of light conditions [79,80,81], even if this mechanism is largely discussed due to the turbid aquatic environment and it should be confirmed by behavioural experiments [83,84].

Besides their eyes, octopus can detect light to trigger the animal’s colour changes using other visual senses. In fact, they can even perceive light through the skins [85], and they can camouflage with the high-fidelity colour to natural and artificial backgrounds [86,87,88,89,90,91]. 

Experiments for testing visual discrimination have been established in octopus [92]. For example, they can quickly learn to visually discriminate between a series of objects [49,51], learn to use vision to direct an arm to a target [47], and recognise familiar conspecifics using vision [48,93]. Octopuses could be visually oriented as well, learning to use visual cues to choose and memorise a den, and take the correct route to return to it [48]. 

When both chemical and visual information is available, octopuses combine information from all sensory inputs that they perceive and then the animals can camouflage themselves, escape a predator, or chase prey in the wild, or open jars for food in captivity [32,94,95]. This integration of several sensory inputs may occur at central and/or peripheral levels [74,96], but the relative contribution of each sense remains poorly understood. 

Our study investigated the priority given to chemical vs. visual perception to establish the sensorial hierarchy in food choice by *O. vulgaris.*

## 2. Materials and Methods 

### 2.1. Animals

Specimens of *O. vulgaris* (n = 4, bodyweight 600 ± 50 g, mean ± SD) were collected from the Bay of Naples (Italy) between June 2018 and October 2018. The animals were transferred to the Di Cosmo’s cephalopod facility at the Department of Biology, University of Naples Federico II, Italy and kept individually in large fibreglass tanks (50 × 50 × 50 cm) filled with seawater [97,98]. Water temperature was kept at 18 ± 1 °C (mean ± SD), and illumination was maintained with natural photoperiod using LED tubes as a light source. All tanks were enriched by adding an amphora (as a den) and rocks (two rocks, about 6 cm^3^). An acclimation period of 15 days was initiated before any experiments were performed. During this time, octopuses were fed ad libitum with crabs (*Carcinus sp*.), a different type of food than was used during trials to reduce the effects of repeated exposure on food choice. The experiments in the present study were conducted in accordance with the principles and procedures that were approved by the Institutional Animal Care of the University of Napoli Federico II and the Ministry of Health (Project n° 608/2016-PR-17 June 2016; protocol n. DGSAF 0022292-P-3 October 2017), and according to the Italian and European law (European Directive 2010/63 EU L276; Italian DL. 4 March 2014, no. 26; the ethical principles of Reduction, Refinement and Replacement).

### 2.2. Experimental Design

**Figure 2:**
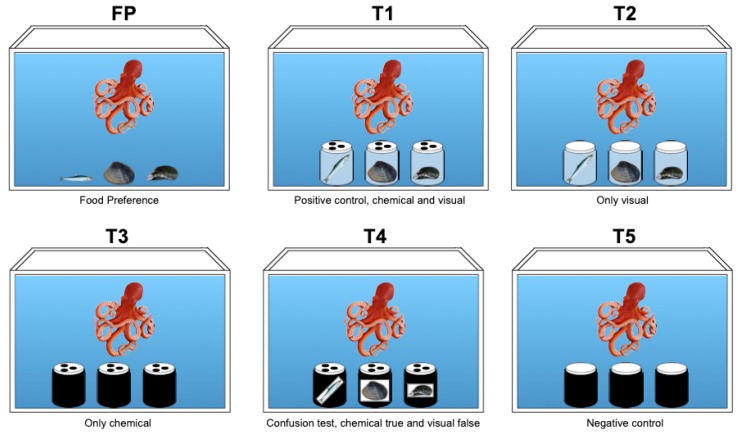
Experimental design to establish the sensorial hierarchy in food choice in *Octopus vulgaris* between chemical and visual cues. Food preference test (**FP**); food types provided in the transparent screw-jars with pierced lids (**T1**), positive control, both chemical and visual cues; food types in transparent screw-jars with no-pierced lids (**T2**), only visual cues; food types in blind screw-jars with pierced lids (**T3**), only chemical cues; food types in screw-jars with pierced lids with outside a photo of food (anchovies, clam, mussel) that is different from the food inside (**T4**), chemical true and visual false cues; food types inside blind screw-jars with no-pierced lids as control (**T5**), negative control, both chemical and visual cues are absent.

To establish the priority given to chemical vs. visual cues in food choice, we defined a behavioural experimental design (Figure 2). 

Firstly, we tested the octopus food preference (FP), giving them three different food types (anchovy, *Engraulis encrasicolus*; clam, *Ruditapes philippinarum*; mussel, *Mytilus edulis*) for 7 days. All foods were placed within octopus’s visual field at the same distance and simultaneously. In the FP test, we evaluated the first food eaten among three provided that should correspond to the favourite one.

Then, to investigate an octopus’s ability to identify the jar containing their favourite food, we subjected the octopuses to five problem-solving tasks (T1, T2, T3, T4, T5, Figure 2, Figure S1):

- T1 (positive control)—food provided in transparent screw-jars with pierced lid;
- T2—food provided in sealed (not pierced) and transparent screw-jars;
- T3—food provided in no-transparent (blind) screw-jars with pierced lid;
- T4 (confusion task)—food provided in the blind screw-jars with pierced lid supplied outside with a realistic picture of the food that results different from what is inside;
- T5 (negative control)—food provided in completely blind and sealed screw-jars.

Each octopus was exposed to 5 trial days for each task. All experiments (FP, T1–T5) were conducted once per day and recorded for at least 1 hr with a digital camera (GoPro Hero 5) positioned on the front of the aquarium (20 cm), to analyse octopus’s choice and behavioural responses, such as exploring, selecting the jar, and eating. We performed FP followed by T1 as first, then animals were tested with the tasks fromT2 toT5 randomly. 

In the FP tests, we considered the food that was eaten, while in the tasks we considered four behaviours (1, jar touched; 2, jar opened; 3, food touched; 4, food eaten) and the time that animals spend to choose the jar to open, from the very first touch to the grab and wrap of the jar starting to open it (Δt).

### 2.3. Statistical Analysis

We examined videos using a high-resolution media player (QuickTime 7, Apple Inc., Cupertino, CA, USA) for behavioural analysis and we recorded data into an Excel data sheet (Microsoft Excel 15.32). Data are expressed in percentages and to analyse the data, we used GraphPad Prism 8 software, SPSS (IBM Corp., Armonk, NY, USA) and R cran [99], performing the Friedman and Wilcoxon matched-pairs tests on ranks within and between experimental conditions. 

## 3. Results

### 3.1. Food Preference in O. vulgaris

During acclimatisation, animals readily recovered their normal behavioural repertoire and did not show any sign of distress. During the food preference test (FP), octopuses approached and explored the different food items presented (Table 1). Animals readily grabbed and fed based on their individual choice. All octopuses touched the three kinds of food provided, exhibiting no significant differences in the first touch for the proposed foods (Table 1, Figure S2, Table S1). 

Although, evaluating the first food eaten, octopuses showed a significative preference for anchovies as a first choice (high-preference), followed by clams (moderate-preference), and mussels (low-preference; Table 2 and Table 3, Figure S2, Table S2).

### 3.2. Food Choice under Different Problem-Solving Tasks

In five of discrimination tasks (T1–T5), jars containing different foods were placed into the bottom of the tank, and all animals performed behaviours such as touching, exploring, and opening the jars. The results revealed that octopuses did not show significant differences in jar-touching behaviours among any of the tests (Friedman test T1–T5, Chi-square = 38.460, *p* = 0.000). Conversely, they generally showed high significant variance in food recognition ability associated with the jar-opening task during all discrimination experiments (Table 4 and Table 5, Figure S3, Table S3). In T1 (both chemical and visual discriminations are available), octopuses revealed the most significant ability to distinguish the food inside the jars and subsequently open the jar that contained the preferred food (anchovies), then the mussel, whereas the clam was ignored. In T2 (only visual discrimination), the octopuses had a significant decrease in recognition of the jar that contained the preferred food. In T3 (only chemical discrimination), the octopuses showed a highly significant difference in selecting the jar containing the preferred food. In the confusion task T4 (real chemical and false visual cues), the octopuses had to overcome this confusion effect by following one of the two cues to find the right jar containing the preferred food. In T4, octopuses exhibited high significant ability in food recognition guided by chemoreception in the opening selected jar containing anchovies. In the control group (T5), without any possibility to distinguish the food inside the jars neither by vision nor by chemosensory cues, all octopuses showed no difference in choosing the jar.

### 3.3. Food Preference Compared to Jar Choice under Different Problem-Solving Tasks 

We analysed the octopus’s food preference considering only food eaten, after jar opening (Table 6 and Table 7, Table S4). Octopuses exhibited no significant difference in the first eaten food comparing FP to each discrimination task, in fact, the first choice was always anchovies, the second clams, and then mussels. In T1, in which octopuses can use both chemical and visual cues, the first food eaten resulted in 100% anchovies, ignoring clams and mussels. Similarly, in T2, in which animals can use just visual cues, the first food eaten resulted in 100% anchovies as above. In T3, where animals perceived just chemical cues, the first food eaten resulted in anchovies, followed by clams, and ignoring mussels. Later, in T4, in which octopuses perceived the right chemical cues while the visual one was false, the first food eaten resulted in 100% anchovies, avoiding clams and mussels. In the control task, where octopuses were not allowed to perceive either visual or chemical cues, the choices among the three food were randomly directed. 

### 3.4. Time Spent to Choose Which Jar Opening under Different Problem-Solving Tasks

**Figure 3:**
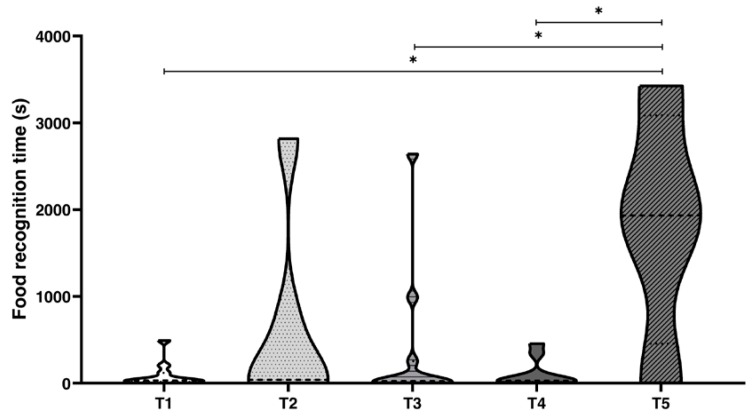
Violin plot of the time spent (Δt) by *Octopus vulgaris* in problem-solving. T1 combined chemical and visual discriminations; T2 only visual discrimination; T3 only chemical discrimination; T4 real chemical and false visual discriminations; T5 negative control. Δt (s): time spent to choose the jar to open, from the very first touch to the grab and wrap of the jar starting to open it. Wilcoxon matched-pairs test vs T1: significance is denoted with asterisks * for *p* < 0.05.

To elucidate which sense was most important in food choice, we compared the time spent to recognise the preferred food inside the jar using visual and chemical perceptions, combined or separately (Figure 3, Table 8 and Table 9, Table S5). We considered the time spent by an octopus from the very first touch and choice of the jar to open (Δt, Figure 3). All octopuses opened the jars to reach the food independently from the task proposed, and showed relatively differences in time needed to make the choice (Figure 3). Octopuses spent a few seconds to recognise the preferred food when chemical and visual cues were available for T1 (Δt (s) = 31.0, Me), while the time increased considerably when one of the two or both senses were limited; in fact, for T2 (visual cues only), Δt (s) = 394.50 s (Me); in T3 (chemical cues only), Δt (s) = 25.00 s (Me); in T4 (true chemical and false visual cues), Δt (s) = 22.50 s (Me); and finally, in T5 (negative control), Δt (s) = 1932.00 (Me). 

## 4. Discussion

In the wild, octopuses are generalist and opportunistic predators that prey on a great variety of species [100,101]. There were strong differences in prey preference among individual octopuses and the prey choice could be varied according to several factors such as predation risks, interspecific competition, or local prey abundance [102]. 

In captivity, octopuses have shown preferences for selected preys [103,104,105], making their food choice by using their sophisticated sense organs. Among these, much attention has been given to vision [22,79,80,81,82]. They also possess sensitive olfactory organs that they use to detect chemicals in the water [23,57,106,107]. However, they also possess suckers that have excellent tactile and chemical sensitivity to perceive chemicals by touch [55,62,68]. Both chemical and visual information is elaborated and stored in specific brain lobes, located in the supra-oesophageal mass and optic lobes [17,92]. Nevertheless, no previous study has addressed the question on which sense has the priority in the food search and choice, although most of the behavioural studies, performed prior to this one, were focused on just their visual capabilities [47,51,64,108,109,110,111,112]. Chemical perception is undoubtedly the first sense that evolved, resulting in widespread sensory modality in all animals. The biological system generated an enormous number of receptors genes to detect and recognise chemicals, but chemoperception resulted largely underappreciated by scientists, even in light of sensory drive evolution theory [113,114].

Here, we establish the priority given to chemical *versus* visual perception in octopus’s food choice just using the ethological approach. Our experiments are performed on a small animal group made by four samples that allow us to investigate the octopus’s behaviour, in according with the 3Rs rules (reduction, refinement, replacement) as allowed by the Italian law (European Directive 2010/63 EU L276; the Italian DL. 3 April 2014, no. 26).

On the basis of the octopus’s exploratory behaviours observed during food preference test (FP), animals tested show a clear preference for the anchovies. However, the first food touch was not always consistent with the food preference (Figure 3), exhibiting a peculiar exploratory behaviour when they approach a new environment [80,94,103,115,116,117,118,119]. Although the fact that octopuses did not touch the preferred food immediately is a clear sign that they cannot rely just on visual perception when they approach a prey, evidently, they need to acquire more information about what they see, using other senses like chemical and tactile, to understand the nature of what they are going to eat. 

To this end, octopuses are equipped with arms containing a widespread chemotactic sensory system concentrated in the hundreds of suckers [24,54,61,63,120,121]. Thus, food choice in octopuses is driven by multiple sensory cues; nevertheless, a hierarchy in sensory perceptions could be hypothesised. 

In our experiments, it was clear that they are mainly attracted by the physical presence of it, without recognising the preferred food by vision at distance. Subsequently, after a random first touch (Figure 3), octopuses start an evaluation of the food using tactile and chemical senses. This allowed us to recognise a temporal hierarchy, where the octopus uses first visual, tactile, and chemical senses, in this order. Our observations are in agreement with previous studies in which it has been reported that octopuses are visually oriented towards a new given object, and then explored it with their arms [103,110,122]. In this case, we are not able to define which sensory cues are dominant by which they arrive at a decision on food choice, because this behaviour represents the result of the integration of different sensory information coming from visual, tactile, and/or chemical systems sequentially to perform a suitable behaviour. 

To understand whether there is a sensorial hierarchy between visual and chemical, to establish which is dominant in decision-making, we tested octopuses with five discrimination tasks. Problem-solving and flexible tool-use are considered hallmarks of cognitive abilities and intelligence [123,124]. In the wild, octopuses exhibit behavioural flexibilities in solving many kinds of problem. For instance, the giant octopus, while attached to a rock can use one of the arm tips to attract a seagull, then when the seagull gets close the sea surface and within the range of the animal’s arm, it grabs and draws the bird into the water (https://youtu.be/LNwegprmtx8). In captivity, octopus also exhibits cognitive abilities in solving problems, when challenged with artificial tasks. Octopuses could retrieve L-shaped food containers from crevices, with or without visual access and independently from the spatial orientation of containers [32] or learn how to unscrew a jar to reach the food [95]. The data here discussed clearly show the ability of octopuses to open jars during all five discrimination tasks successfully. 

Our findings indicate that octopuses recognise the jar containing the anchovy, that resulted to be the preferred food in FP test (Table 2 and Table 3), in all discrimination tasks (T1–T4), with the exception of the negative control (T5) (Table 4 and Table 5). However, the task in which it is evident that the dominant sense is the chemical one, it is the confusion task (T4), where, despite the fact that octopuses were cheated with a false picture of the food inside, they picked up anchovy in the 100% of the cases. This evidence is corroborated when we excluded the chemical cues, focusing exclusively on the visual sense (T2), in which the jar containing anchovy was selected in only 50% of the cases. The negative control experiment (T5) reinforces our claim, in fact, that the chemical and visual information was not used by octopuses in solving this task, so the choice was randomly made.

These findings are consistent with Mather and collegues [125] recently reporting that octopuses did not open the jar to get a small crayfish inside, because chemical cues from herring were smeared on its surface. Our experimental design (Figure 2) allowed us to demonstrate that in *O. vulgaris* both chemical and visual perceptions are essential in food choice; nevertheless, the chemical signals are the most important inputs. On the other hand, when we compared food eaten to jar choice under all different problem-solving tasks, we discovered that the preference to anchovies was maintained, even when the first jar chosen was not containing the anchovy (Table 4 and Table 5). In fact, in T2, where octopuses could just see the food inside, in 33.3% of cases, they opened first the jar containing a mussel, but because octopuses do not eat it, they resultantly were forced to look for another “chance to win” the preferred food. Merging the data coming from all tasks (Table 4 and Table 5), we observed that the percentage of successful decisions to open as first the jar contained the preferred food, based on chemical cues, were significantly higher than visual one (88.9% vs. 11.1% respectively, Wilcoxon matched-pairs test *p* < 0.05). 

**Figure 4:**
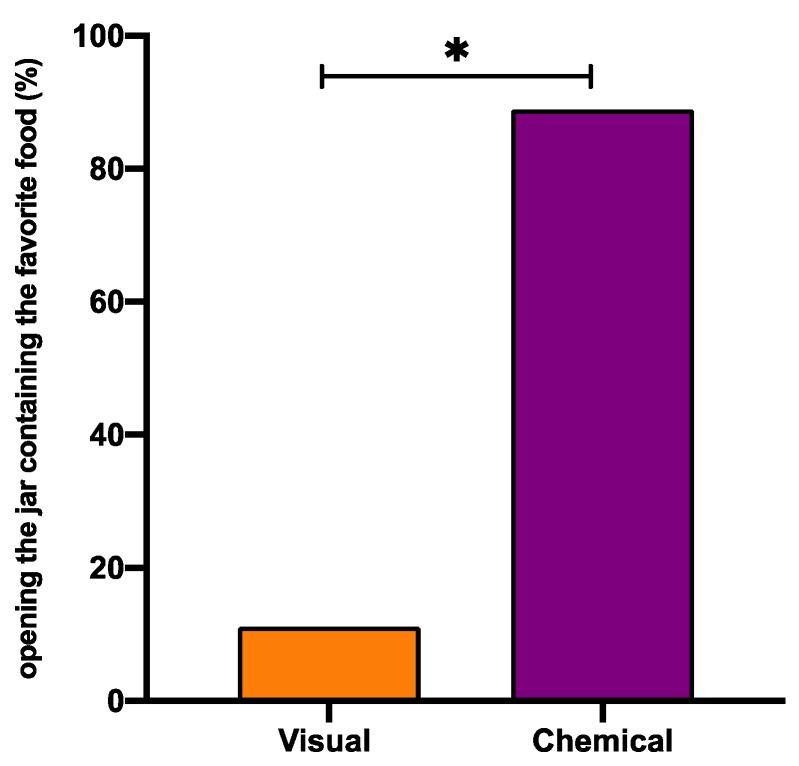
Visual vs. chemical perception in *Octopus vulgaris*. Percentage of the successful decisions to open as first the jar that contained the preferred food, based on chemical (purple) or visual (orange) cues. Wilcoxon matched-pairs test vs. T1 significance is denoted with asterisks * for *p* < 0.05.

Furthermore, when we considered Δt, octopuses spent more time to visually discriminate the preferred food than either by combined visual and chemical discrimination or by chemical discrimination only (Figure 4). Despite the fact that the differences encountered in Δt are affected by inter-individual variability, that is well known in this animal, and the limited number of specimens used, these results indicate that octopuses are able to decrease Δt to correctly solve operant tasks based on chemical information (Figure 4), this might be of importance in predation strategy in the wild where the prey is not closed in a jar, but hidden and ready to escape. However, while we posit that octopus, privileges its chemical stimuli over visual ones, we should appreciate that the two combined increase the probability of success in prey [74,95,96,126].

Our results demonstrate involvement of chemosensory sense in octopus food choice behaviour, and allows a reassessment of the importance of chemical perception in the ecology of octopus. Octopus is, in fact, able to detect chemical cues with different spatial ranges through either contact or distant chemoreception. Its capability is based on the presence of the olfactory organ, structure mainly dedicated for hydrosoluble molecules, and the numerous chemoreceptors bared on its sucker rims that are essential to perceive and explore bi-dimensional traces consisting of insoluble molecules released on the seafloor in turn by preys, predators, or conspecifics [74,75]. These considerations will open new perspectives to study the behaviour of such an intriguing animal that is the octopus.

## Figures and Tables

**Table 1:** First touch during the food preference test (percentage).

Food	Me	IQR (Q1; Q3)	Friedman Test (Chi-Square; *p*-Value)
Anchovies	33.33	25.00; 55.00	1.524; 0.467
Clams	33.33	29.16; 45.00	
Mussels	33.33	12.50; 40.00	

Median (Me), Interquartile Range (IQR): Q1—first quartile, Q3—third quartile, Friedman test.

**Table 2:** Food preference test (percentage).

Food	Me	IQR (Q1; Q3)	Friedman Test (Chi-Square; *p*-Value)
Anchovies	66.67	66.67; 90.00	11.120; **0.004**
Clams	20.00	0.00; 33.33	
Mussels	0.00	0.00; 0.00	

Median (Me), Interquartile Range (IQR): Q1—first quartile, Q3—third quartile, Friedman test, *p*-values < 0.05 are marked in bold.

**Table 3:** Food preference test. Wilcoxon matched-pairs test significance *p*-value, *p*-values < 0.05 are marked in bold.

Food	Anchovies	Clams
**Clams**	**0.027**	
**Mussels**	**0.018**	0.216

**Table 4:** Food choice under different tasks in *Octopus vulgaris* (percentage).

Task	Food	Me	IQR (Q1; Q3)	Friedman Test (Chi-Square; *p*-Value)
T1 Combined Chemical and Visual Cues	Anchovies	100.00	75.00; 100.00	8.400; **0.015**
	Clams	0.00	0.00; 0.00	
	Mussels	0.00	0.00; 25.00	
T2 Only Visual Cues	Anchovies	50.00	0.00; 100.00	3.500; 0.174
	Clams	0.00	0.00; 0.00	
	Mussels	0.00	0.00; 75.00	
T3 Only Chemical Cues	Anchovies	100.00	58.33; 100.00	7.625; **0.022**
	Clams	0.00	0.00; 16.67	
	Mussels	0.00	0.00; 25.00	
T4 Real Chemical and False Visual Cues	Anchovies	100.00	75.00; 100.00	8.400; **0.015**
	Clams	0.00	0.00; 0.00	
	Mussels	0.00	0.00; 25.00	
T5 Negative Control	Anchovies	0.00	0.00; 100.00	0.400; 0.819
	Clams	0.00	0.00; 50.00	
	Mussels	0.00	0.00; 100.00	

Median (Me), Interquartile Range (IQR): Q1—first quartile, Q3—third quartile, Friedman test, *p*-values < 0.05 are marked in bold.

**Table 5:** Food choice under different tasks in *O. vulgaris*. Wilcoxon matched-pairs test significance *p*-value, *p*-values < 0.05 are marked in bold.

T1 Combined Chemical and Visual Cues	Anchovies	Clams
**Clams**	**0.034**	
**Mussels**	**0.046**	0.317
**T3 Only Chemical Cues**	**Anchovies**	**Clams**
**Clams**	**0.039**	
**Mussels**	**0.049**	0.655
**T4 Real Chemical and False Visual Cues**	**Anchovies**	**Clams**
**Clams**	**0.034**	
**Mussels**	**0.046**	0.317

**Table 6:** Maintained food preference during problem-solving tasks in *Octopus vulgaris* (percentage).

Task	Food	Me	IQR (Q1; Q3)	Friedman Test (Chi-Square; *p*-Value)
T1 Combined Chemical and Visual Cues	Anchovies	100.00	100.00; 100.00	10.000; **0.007**
	Clams	0.00	0.00; 0.00	
	Mussels	0.00	0.00; 0.00	
T2 Only Visual Cues	Anchovies	100.00	100.00; 100.00	8.000; **0.018**
	Clams	0.00	0.00; 0.00	
	Mussels	0.00	0.00; 0.00	
T3 Only Chemical Cues	Anchovies	100.00	83.33; 100.00	9.500; **0.009**
	Clams	0.00	0.00; 16.67	
	Mussels	0.00	0.00; 0.00	
T4 Real Chemical and False Visual Cues	Anchovies	100.00	100.00; 100.00	10.000; **0.007**
	Clams	0.00	0.00; 0.00	
	Mussels	0.00	0.00; 0.00	
T5 Negative Control	Anchovies	0.00	0.00; 50.00	0.000; 1.000
	Clams	0.00	0.00; 50.00	
	Mussels	0.00	0.00; 50.00	

Median (Me), Interquartile Range (IQR): Q1—first quartile, Q3—third quartile, Friedman test, *p*-values < 0.05 are marked in bold.

**Table 7:** Maintained food preference during problem-solving tasks in *Octopus vulgaris*. Wilcoxon matched-pairs test significance *p*-value.

T1 Combined Chemical and Visual Cues	Anchovies	Clams
**Clams**	**0.025**	
**Mussels**	**0.025**	1.000
**T2 Only Visual Cues**	**Anchovies**	**Clams**
**Clams**	**0.046**	
**Mussels**	**0.046**	1.000
**T3 Only Chemical Cues**	**Anchovies**	**Clams**
**Clams**	**0.034**	
**Mussels**	**0.034**	0.317
**T4 Real Chemical and False Visual Cues**	**Anchovies**	**Clams**
**Clams**	**0.025**	
**Mussels**	**0.025**	1.000

**Table 8:** Time spent (Δt) by *Octopus vulgaris* in problem-solving (second).

Task	Me	IQR (Q1; Q3)	Friedman Test (Chi-Square; *p*-Value)
T1 combined chemical and visual cues	31.00	18.50; 47.25	9.055; 0.059
T2 only visual cues	394.50	35.00; 1263.50	
T3 only chemical cues	25.00	13.00; 30.00	
T4 real chemical and false visual cues	22.50	15.25; 147.50	
T5 negative control	1932.00	1356.00; 2401.00	

Median (Me), Interquartile Range (IQR): Q1—first quartile, Q3—third quartile, Friedman test.

**Table 9:** Time spent (Δt) by *Octopus vulgaris* in problem-solving. Wilcoxon matched-pairs test significance *p*-value, *p*-values < 0.05 are marked in bold.

	T1 Combined Chemical and Visual Cues	T2 Only Visual Cues	T3 Only Chemical Cues	T4 Real Chemical and False Visual Cues
T2 only visual cues	0.117			
T3 only chemical cues	0.944	0.108		
T4 real chemical and false visual cues	0.346	0.442	0.780	
T5 negative control	**0.009**	0.407	**0.009**	**0.029**

## Supplementary Materials

The following are available online at https://www.mdpi.com/2076-2615/10/3/457/s1, Figure S1: frames of *O. vulgaris* under task. Animal body weight 600 g; jar diameter 5.5 cm, height 7.0 cm. Figure S2: First touch and food preference in *O. vulgaris*. (**A**) Boxplot of the food that was touched first (Friedman test, *p* > 0.05); (**B**) Boxplot of food that was eaten first (Friedman test, *p* < 0.05). Wilcoxon matched pairs test significance is denoted with asterisks * for *p* < 0.05. Figure S3: Food choice under different tasks in *O. vulgaris*. T1 combined chemical and visual cues; T2 only visual cues; T3 only chemical cues; T4 real chemical and false visual cues; T5 negative control. (**A**) five day trend preferences; (**B**) single day preferences. Friedman test T1–T5, Chi-square = 38.460, *p* < 0.05. Data were log transformed, Wilcoxon matched pairs test significance is denoted with asterisks * for *p* < 0.05. Table S1: First touch during the food preference test (percentage). Median (Me), Interquartile Range IQR: Q1 first quartile and Q3 third quartile, Mean (M), Standard deviation (SD), Standard error (SE), Friedman test, *p*-values < 0.05 are marked in bold. Table S2: Food preference test (percentage). Median (Me), Interquartile Range IQR: Q1 first quartile and Q3 third quartile, Mean (M), Standard deviation (SD), Standard error (SE), Friedman test, *p*-values < 0.05 are marked in bold. Table S3: Food choice under different tasks in *Octopus vulgaris* (percentage). Median (Me), Interquartile Range IQR: Q1 first quartile and Q3 third quartile, Mean (M), Standard deviation (SD), Standard error (SE), Friedman test, *p*-values < 0.05 are marked in bold. Table S4: Maintain food preference during problem-solving tasks in *Octopus vulgaris* (percentage). Median (Me), Interquartile Range IQR: Q1 first quartile and Q3 third quartile, Mean (M), Standard deviation (SD), Standard error (SE), Friedman test, *p*-values < 0.05 are marked in bold. Table S5: Time spent (Δt) by *Octopus vulgaris* in problem-solving (second). Median (Me), Interquartile Range IQR: Q1 first quartile and Q3 third quartile, Mean (M), Standard deviation (SD), Standard error (SE), Friedman test, *p*-values < 0.05 are marked in bold.
