# Supplementary material for: Sensorial Hierarchy in Octopus vulgaris’s Food Choice: Chemical vs. Visual
Source: Animals (Basel). 2020 Mar 10;10(3):457. doi: 10.3390/ani10030457 (PMC7143185; doi:10.3390/ani10030457)
Supplement: Supplementary file 1 [file animals-10-00457-s001.pdf]

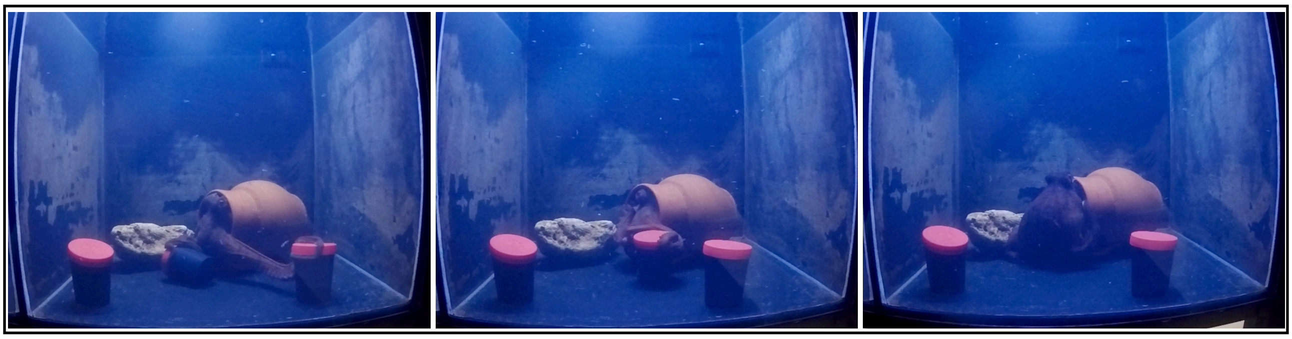

**Figure S1. Video frames of *O.vulgaris* under task.** Animal body weight 600g; jar diameter 5.5 cm, height 7.0 cm.

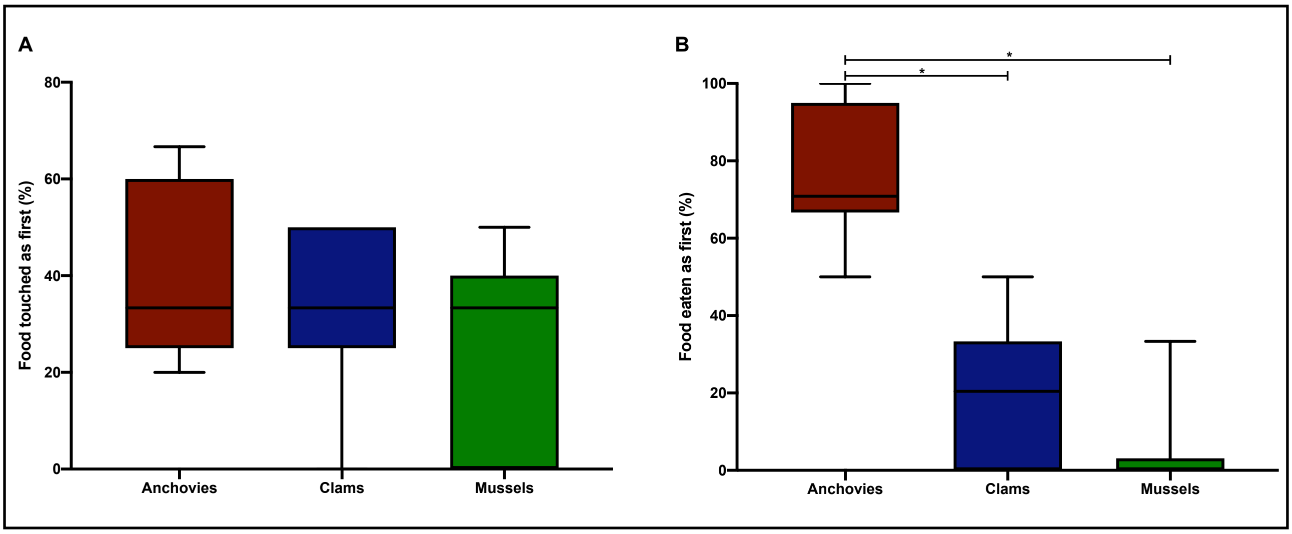


**Figure S2. First touch and food preference in *O. vulgaris*.** (**A**) Boxplot of the food that was touched first (Friedman test, p>0.05); (**B**) Boxplot of food that was eaten first (Friedman test, p<0.05). Wilcoxon matched pairs test significance is denoted with asterisks * for p <0.05.

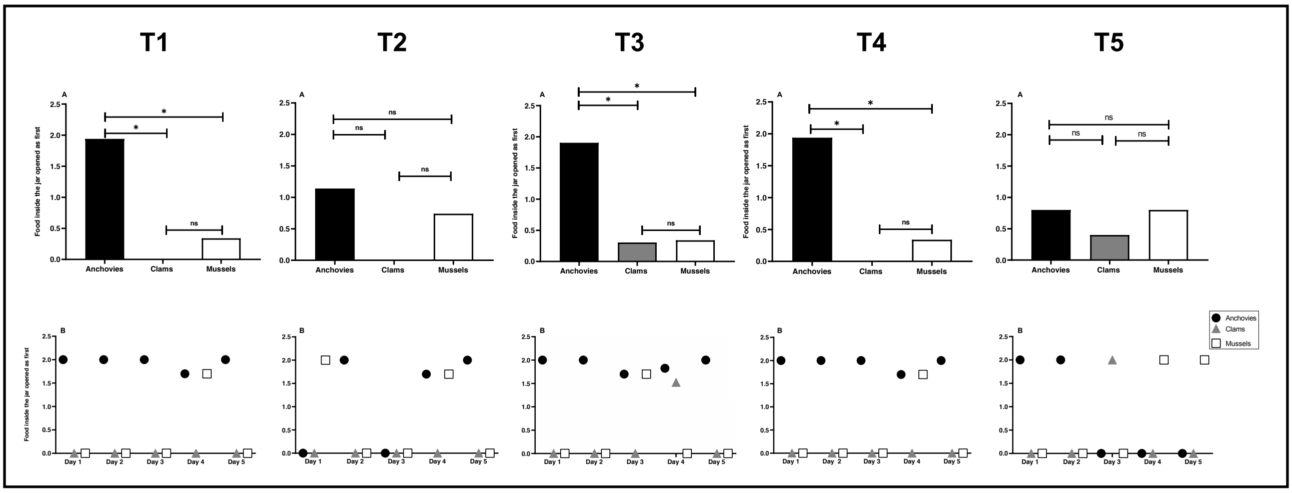


**Figure S3. Food choice under different tasks in *O. vulgaris*.** T1 combined chemical and visual cues; T2 only visual cues; T3 only chemical cues; T4 real chemical and false visual cues; T5 negative control. (a) five day trend preferences; (b) single day preferences. Friedman test T1-T5, Chi-square = 38.460, p<0.05. Data were log transformed, Wilcoxon matched pairs test significance is denoted with asterisks * for p <0.05.

**Table S1 - First touch during the food preference test (percentage).** Median (Me), Interquartile Range IQR: Q1 first quartile and Q3 third quartile, Mean (M), Standard deviation (SD), Standard error (SE), Friedman test.

| | Me | IQR [Q1; Q3] | M | SD | SE | Friedman test [Chi-square; p-value] |
|---|---|---|---|---|---|---|
| **Anchovies** | 33.33 | 25.00; 55.00 | 40.00 | 18.73 | 7.08 | |
| **Clams** | 33.33 | 29.16; 45.00 | 33.09 | 17.22 | 6.51 | 1.524; 0.467 |
| **Mussels** | 33.33 | 12.50; 40.00 | 26.90 | 19.87 | 7.51 | |

**Table S2 - Food preference test (percentage).** Median (Me), Interquartile Range IQR: Q1 first quartile and Q3 third quartile, Mean (M), Standard deviation (SD), Standard error (SE), Friedman test.

| | Me | IQR [Q1; Q3] | M | SD | SE | Friedman test [Chi-square; p- value] |
|---|---|---|---|---|---|---|
| **Anchovies** | 66.67 | 66.67; 90.00 | 75.71 | 18.73 | 7.08 | |
| **Clams** | 20.00 | 0.00; 33.33 | 19.52 | 20.22 | 7.64 | 11.120; **0.004** |
| **Mussels** | 0.00 | 0.00; 0.00 | 4.76 | 12.60 | 4.76 | |

**Table S3 - Food choice under different tasks in *Octopus vulgaris* (percentage).** Median (Me), Interquartile Range IQR: Q1 first quartile and Q3 third quartile, Mean (M), Standard deviation (SD), Standard error (SE), Friedman test.

| | | Me | IQR [Q1; Q3] | M | SD | SE | Friedman test [Chi-square; p- value] |
|---|---|---|---|---|---|---|---|
| *T1 combined chemical and visual cues* | **Anchovies** | 100.00 | 75.00; 100.00 | 90.00 | 22.36 | 10.00 | 8.400; **0.015** |
| | **Clams** | 0.00 | 0.00; 0.00 | 0.00 | 0.00 | 0.00 | |
| | **Mussels** | 0.00 | 0.00; 25.00 | 10.00 | 22.36 | 10.00 | |
| *T2 only visual cues* | **Anchovies** | 50.00 | 0.00; 100.00 | 50.00 | 50.00 | 22.36 | 3.500; 0.174 |
| | **Clams** | 0.00 | 0.00; 0.00 | 0.00 | 0.00 | 0.00 | |
| | **Mussels** | 0.00 | 0.00; 75.00 | 30.00 | 44.72 | 20.00 | |
| *T3 only chemical cues* | **Anchovies** | 100.00 | 58.33; 100.00 | 83.33 | 23.57 | 10.54 | 7.625; **0.022** |
| | **Clams** | 0.00 | 0.00; 16.67 | 6.67 | 14.91 | 6.67 | |
| | **Mussels** | 0.00 | 0.00; 25.00 | 10.00 | 22.36 | 10.00 | |
| *T4 real chemical and false visual cues* | **Anchovies** | 100.00 | 75.00; 100.00 | 90.00 | 22.36 | 10.00 | 8.400; **0.015** |
| | **Clams** | 0.00 | 0.00; 0.00 | 0.00 | 0.00 | 0.00 | |
| | **Mussels** | 0.00 | 0.00; 25.00 | 10.00 | 22.36 | 10.00 | |
| *T5 negative control* | **Anchovies** | 0.00 | 0.00; 100.00 | 40.00 | 54.77 | 24.49 | 0.400; 0.819 |
| | **Clams** | 0.00 | 0.00; 50.00 | 20.00 | 44.72 | 20.00 | |
| | **Mussels** | 0.00 | 0.00; 100.00 | 40.00 | 54.77 | 24.49 | |

**Table S4 - Maintain food preference during problem-solving tasks in *Octopus vulgaris* (percentage).** Median (Me), Interquartile Range IQR: Q1 first quartile and Q3 third quartile, Mean (M), Standard deviation (SD), Standard error (SE), Friedman test.

| Maintain food preference during problem-solving tasks in O. vulgaris _*Figure 5* | | | | | | | |
|---|---|---|---|---|---|---|---|
| | | **Me** | **IQR [Q1; Q3]** | **M** | **SD** | **SE** | **Friedman test [Chi-square; p- value]** |
| *T1 combined chemical and visual cues* | **Anchovies** | 100.00 | 100.00; 100.00 | 100.00 | 0.00 | 0.00 | 10.000; **0.007** |
| | **Clams** | 0.00 | 0.00; 0.00 | 0.00 | 0.00 | 0.00 | |
| | **Mussels** | 0.00 | 0.00; 0.00 | 0.00 | 0.00 | 0.00 | |
| *T2 only visual cues* | **Anchovies** | 100.00 | 100.00; 100.00 | 80.00 | 44.72 | 20.00 | 8.000; **0.018** |
| | **Clams** | 0.00 | 0.00; 0.00 | 0.00 | 0.00 | 0.00 | |
| | **Mussels** | 0.00 | 0.00; 0.00 | 0.00 | 0.00 | 0.00 | |
| *T3 only chemical cues* | **Anchovies** | 100.00 | 83.33; 100.00 | 93.33 | 14.91 | 6.67 | 9.500; **0.009** |
| | **Clams** | 0.00 | 0.00; 16.67 | 6.67 | 14.91 | 6.67 | |
| | **Mussels** | 0.00 | 0.00; 0.00 | 0.00 | 0.00 | 0.00 | |
| *T4 real chemical and false visual cues* | **Anchovies** | 100.00 | 100.00; 100.00 | 100.00 | 0.00 | 0.00 | 10.000; **0.007** |
| | **Clams** | 0.00 | 0.00; 0.00 | 0.00 | 0.00 | 0.00 | |
| | **Mussels** | 0.00 | 0.00; 0.00 | 0.00 | 0.00 | 0.00 | |
| *T5 negative control* | **Anchovies** | 0.00 | 0.00; 50.00 | 20.00 | 44.72 | 20.00 | 0.000; 1.000 |
| | **Clams** | 0.00 | 0.00; 50.00 | 20.00 | 44.72 | 20.00 | |
| | **Mussels** | 0.00 | 0.00; 50.00 | 20.00 | 44.72 | 20.00 | |

**Table S5 - Time spent (Δt) by *Octopus vulgaris* in problem-solving (second).** Median (Me), Interquartile Range IQR: Q1 first quartile and Q3 third quartile, Mean (M), Standard deviation (SD), Standard error (SE), Friedman test.

| | Me | IQR [Q1; Q3] | M | SD | SE | Friedman test [Chi-square; p-value] |
|---|---|---|---|---|---|---|
| ***T1 combined chemical and visual cues*** | 31.00 | 18.50; 47.25 | 52.1 | 62.96 | 19.91 | 9.055; 0.059 |
| ***T2 only visual cues*** | 394.50 | 35.00; 1263.50 | 904 | 1320.97 | 660.49 | |
| ***T3 only chemical cues*** | 25.00 | 13.00; 30.00 | 151.78 | 324.05 | 108.02 | |
| ***T4 real chemical and false visual cues*** | 22.50 | 15.25; 147.50 | 120.25 | 176.43 | 62.38 | |
| ***T5 negative control*** | 1932.00 | 1356.00; 2401.00 | 1824.5 | 1407.11 | 703.55 | |
